# Exploring Potential Therapeutic Applications of Tazarotene: Gene Regulation Mechanisms and Effects on Melanoma Cell Growth

**DOI:** 10.3390/cimb47040237

**Published:** 2025-03-28

**Authors:** Chun-Hua Wang, Lu-Kai Wang, Fu-Ming Tsai

**Affiliations:** 1 Department of Dermatology, Taipei Tzu Chi Hospital, Buddhist Tzu Chi Medical Foundation, New Taipei City 231, Taiwan; dermawang@gmail.com; 2 School of Medicine, Tzu Chi University, Hualien 970, Taiwan; 3 Operation & Promotion Division, National Center for Biomodels, National Institutes of Applied Research, Taipei City 115, Taiwan; 2407026@narlabs.org.tw; 4 Department of Research, Taipei Tzu Chi Hospital, Buddhist Tzu Chi Medical Foundation, New Taipei City 231, Taiwan

**Keywords:** tazarotene, retinoic acid, melanoma, tazarotene-induced gene, retinoic acid receptors, retinoid X receptors

## Abstract

Tazarotene, a retinoid derivative, is widely used in treating skin conditions such as psoriasis and acne. Recent studies have demonstrated its potential as a promising therapeutic agent for treating melanoma in situ. Its primary mechanism of action involves the selective activation of retinoic acid receptors (RAR-β and RAR-γ), which play important roles in regulating cell growth, differentiation, and apoptosis. By activating these receptors, tazarotene influences the expression of several downstream inducible genes, such as tazarotene-induced gene-1 (*TIG1*), *TIG2*, and *TIG3*. These genes play crucial roles in regulating melanoma cell proliferation, invasiveness, and immune responses in the tumor microenvironment. This review aims to provide a comprehensive overview of the current status of retinoid derivatives—particularly tazarotene—in melanoma treatment and the latest research regarding their molecular mechanisms. We will explore how tazarotene suppresses melanoma growth through gene regulation mechanisms and discuss its potential role in immune responses within the tumor microenvironment. Additionally, we assess the advantages and challenges of using tazarotene as a topical treatment and explore its future clinical applications. These studies contribute to a wider understanding of tazarotene’s antitumor mechanisms, providing a solid theoretical foundation for its potential as a therapeutic option for melanoma in situ.

## 1. Introduction

Cutaneous melanoma is a malignant cancer originating from melanocytes; it causes approximately 55,500 deaths annually, accounting for 0.7% of all cancer-related deaths [1,2]. It is classified into melanoma in situ and metastatic melanoma. The former refers to cancer cells that are confined to the epidermis without invading the dermis. When detected early and surgically excised, melanoma in situ is usually curable [3]. Lentigo maligna melanoma (LMM), a subtype of melanoma in situ, typically occurs in elderly individuals with long-term sun exposure, particularly on the face [4,5]. It presents as irregularly bordered brown or black patches, which, if left untreated, may progress to invasive melanoma [5]. Metastatic melanoma develops when cancer cells spread to lymph nodes or distant organs, such as the lungs, liver, or brain [6]. This stage has a poor prognosis and requires a combination of immunotherapy, targeted therapy, or chemotherapy [7]. Due to melanoma’s aggressive nature and rapid progression, early detection and treatment are crucial for improving survival rates.

Melanoma in situ is typically curable through surgical excision [8]. For larger lesions or those in difficult-to-remove areas, Mohs surgery or topical imiquimod may be used as alternatives [9,10,11]. Metastatic melanoma is more complex to treat due to its spread, often requiring a combination of therapies to improve survival. In recent decades, aside from BRAF inhibitors and mitogen-activated protein kinase (MAPK) inhibitors—which have been shown to extend overall survival—no other effective treatments have emerged, and most clinical trials for advanced melanoma have failed [12,13,14,15]. Large-scale studies [16] and database analyses [17] suggest that retinoic acid (RA) can prevent over 40% of melanoma formation. However, as its side effects limit its use as a therapeutic drug, RA is currently only used as a dietary supplement. Therefore, identifying RA-regulated genes that can reduce these side effects and serve as potential treatment targets may advance melanoma therapies.

**Figure 1:**
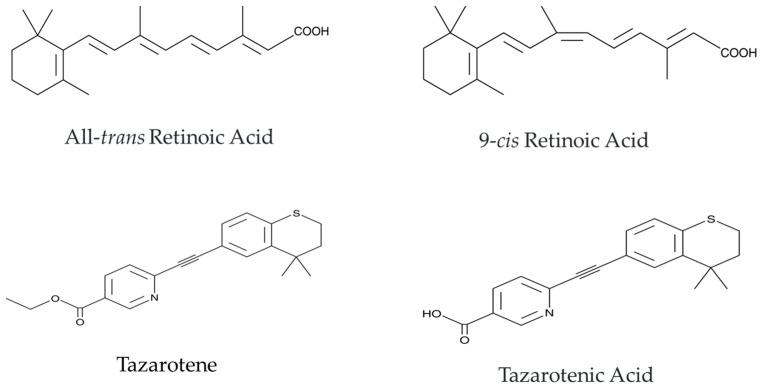
Chemical structures of ATRA, 9-cis-RA, tazarotene, and tazarotenic acid. These 2D structures are taken from Caymanchem. Available online: https://www.caymanchem.com/products/categories, accessed on 14 January 2025. ATRA, all-trans retinoic acid; 9-cis-RA, 9-cis retinoic acid.

RA, the active metabolite of vitamin A, includes biologically active forms like all-trans RA (ATRA) and 9-cis RA (Figure 1) [18,19,20]. RA binds to nuclear RA receptors (RARs) and retinoid X receptors (RXRs), activating RA response elements in DNA to regulate gene transcription. This process affects cell proliferation, differentiation, and apoptosis [21,22,23]. By regulating genes, RA promotes differentiation, limits excessive proliferation, and modulates immune responses, making it crucial for treating skin diseases like acne and psoriasis [24,25,26]. Additionally, in some cases, ATRA can induce cancer cell differentiation or apoptosis and thus inhibit tumor growth [27,28,29,30]. For instance, ATRA has been used to treat acute promyelocytic leukemia by inducing leukemic cells differentiation into mature blood cells [31,32]. However, since RA has an affinity for RAR-α, RAR-β, and RAR-γ receptors, it lacks specificity. Consequently, its use often leads to severe side effects for patients, such as skin irritation (redness and peeling) and more severe systemic side effects, including liver damage, teratogenicity, and neurological issues [33,34,35]. Moreover, because of its simple molecular structure, poor stability, narrow therapeutic index, associated toxicities, —including teratogenic effects—and a long half-life of up to 120 d, the clinical use of RA is greatly limited [36].

Tazarotene is a third-generation synthetic retinoid for topical use [37], modified for greater selectivity and stability [38]. As a prodrug, it is metabolized in the skin to its active form, tazarotenic acid (Figure 1) [37,39]. Unlike ATRA, which binds both RAR and RXR receptors, tazarotene mainly targets RAR-β and RAR-γ, avoiding RXR binding. This selectivity helps minimize systemic side effects. The major RARs in the skin are RAR-γ (approximately 87%) and RAR-α [40], which makes tazarotene particularly effective as a topical treatment. After application, tazarotene is quickly converted to tazarotenic acid (half-life 2–18 min) and excreted within 1–2 h in all animal studies [37]. Tazarotene does not exhibit toxicity in CHO cells or keratinocytes; even after conversion to active tazarotenic acid, its toxicity in CHO and keratinocytes was 50 and 10 times lower, respectively, than that of ATRA [41]. In rats and minipigs, chronic local tazarotene application over the course of 1 year did not result in any systemic toxic effects [37].

Applied as a gel or cream, tazarotene selectively activates RAR-β and RAR-γ to suppress abnormal skin cell growth and promote cell differentiation [42,43,44]. However, its oral safety, pharmacokinetics, and efficacy remain unverified, and there are no approved oral formulations. Research in this area is limited. Recent studies on tazarotene-induced gene (*TIG*) regulation (*TIG1*, *TIG2*, *TIG3*) highlight its potential for treating localized melanoma. These genes influence tumor cell proliferation, differentiation, apoptosis, and immune responses. This review explores tazarotene’s gene regulatory effects and its role in melanoma cell growth, providing a foundation for its potential clinical application.

## 2. Tazarotene’s Mechanism of Action

**Figure 2:**
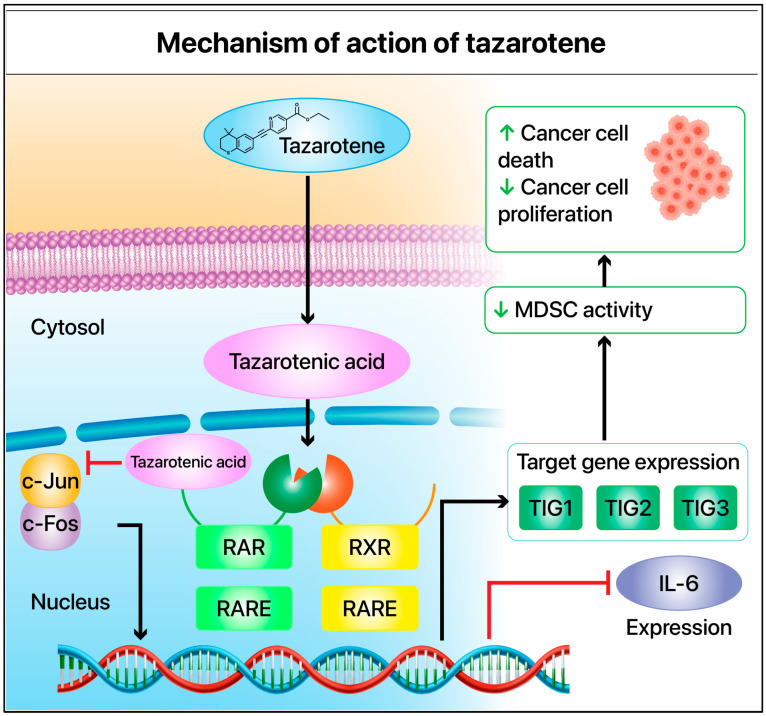
Tazarotene’s mechanism of action. After entering the cell, tazarotene is converted into tazarotenic acid. It then enters the cell nucleus and binds to retinoic acid receptor (RAR), acting as an antagonist of AP1(c-jun/c-fos) and inhibiting the transcription of related genes. Alternatively, it binds to the retinoic acid response elements region of DNA and participates in the transcription of target genes, such as *TIG1*, *TIG2*, and *TIG3*. TIG—tazarotene-induced gene.

Although tazarotene may interact with RXRs, its primary action occurs via the RAR pathway [40,45]. By binding to RAR-β/γ, it activates RA response elements on DNA and antagonizes AP1 and type I interferons. Composed of c-Jun and c-Fos, AP1 is induced by mitogens or oncogenes and promotes cell proliferation and inflammatory responses [46,47]. Histological staining shows that tazarotene inhibits AP1 activity in keratinocytes and reduces inflammatory molecules like interleukin-6 (IL-6) (Figure 2) [43].

Three major genes are induced in keratinocytes treated with tazarotene: *TIG1*, *TIG2*, and *TIG3* (Figure 2) [43,48,49,50,51]. TIG1 is exclusively induced by tazarotene or other RAR-specific retinoids, but not by RXR-specific retinoids, indicating that *TIG1* is mainly activated through the RAR rather than the RXR pathway [52,53]. *TIG1* expression is influenced by the methylation of its promoter and CpG islands, and its expression is often reduced in various cancer tissues [54,55,56,57]. *TIG2* is a chemotactic protein that serves as a ligand for the G protein-coupled receptor CMKLR1 (also known as ChemR23) [58]. *TIG2* can stimulate dendritic cells chemotaxis and macrophages to inflammatory sites [59,60]. *TIG3* is a tumor suppressor gene that mediates cell growth regulation by retinoids [51,61,62,63].

## 3. Prevention and Clinical Management of Melanoma with RA and Tazarotene

An early largescale prospective cohort study investigated the relation between vitamin A and carotenoid intakes and the risk of melanoma. The study found that dietary intake of retinol (the active form of vitamin A) was significantly associated with a reduced risk of melanoma [16]. Furthermore, the results of database analyses have also revealed an association between vitamin A intake and reduced risk of melanoma [17]. However, excessive intake of vitamin A may lead to toxicity, and dietary factors are only one of many factors that influence melanoma risk. Therefore, determining the optimal vitamin A intake and sources requires further research. Additionally, a study by Marloes Helder and colleagues used Mendelian randomization to explore the causal relation between serum retinol levels and the risk of skin cancer, including melanoma. The study concluded that there was no significant association between the two, suggesting that serum retinol levels may not be a protective factor against skin cancers [64]. Therefore, whether daily vitamin A intake can effectively prevent melanoma requires further clinical trials for validation.

In terms of managing melanoma, a study by Wei Yin and colleagues explored the inhibitory effect of topical ATRA on melanoma in mice and found that this mechanism was related to the activation of CD8^+^ T cells in vivo. They discovered that ATRA increased the expression of major histocompatibility complex class I on tumor cells, eliminated myeloid-derived suppressor cells (MDSCs), and promoted their differentiation, thereby enhancing the antitumor immune response in mice [65]. MDSCs play critical roles in melanoma progression. When they accumulate in melanoma tissues and the bloodstream, they suppress the immune system and prevent it from attacking cancer cells [66,67,68]. As such, a higher number of MDSCs in the tumor microenvironment correlates with a poorer prognosis [66]. The ability of ATRA to inhibit MDSC activity has also been investigated in clinical trials involving metastatic melanoma treatment (ClinicalTrials.gov ID: NCT03200847). In a Phase I/II clinical trial conducted by Tobin et al., the combination of ATRA and the immune checkpoint inhibitor (ICI) pembrolizumab showed good safety and significant antitumor activity in metastatic melanoma. In this trial, 24 participants diagnosed with stage M1a or M1b disease received 150 mg/m^2^/d ATRA plus 200 mg pembrolizumab. The results showed a median progression-free survival of 20.3 months, with an overall response rate of 71%. Complete remission was achieved in 50% of patients, and the 1-year overall survival rate was 80% [69]. Moreover, they found that the combination treatment effectively reduced circulating MDSC numbers, suggesting that ATRA may enhance the efficacy of immunotherapy by targeting MDSC differentiation.

In addition to tazarotene, there are other selective drugs for RAR-β (such as Bexarotene) and RAR-γ (such as Ro40-6055 [70]) under development. Bexarotene is mainly used in cancer treatment, particularly for lymphoma [71,72,73]. It regulates tumor cell growth and differentiation by activating RAR-β, showing strong anti-tumor effects. Common side effects of Bexarotene include elevated cholesterol and triglyceride levels, which may lead to liver issues [74,75,76]. On the other hand, selective RAR-γ drugs are largely in the clinical research stages, with relatively limited data on their side effects. Therefore, based on the severity of side effects and its range of action, tazarotene is more commonly used clinically, especially in treating psoriasis and other skin diseases.

In contrast to malignant metastatic melanoma, a study by Galina Shistik et al. reported a case of local metastatic melanoma in an 83-year-old woman treated with a combination of the immune modulator imiquimod and topical tazarotene. After 6 weeks of combined treatment, complete clinical clearance was seen in the treated area [77]. Lentigo maligna (LM) is a common melanoma in situ found on the faces of older adults, for which surgical excision is usually the standard treatment [78,79]. Topical drug therapy may be an alternative treatment for patients who are unsuitable for surgery. A study by Sergio Chimenti et al. highlighted two successful cases of LM treatment with 0.1% tazarotene gel. The patients were administered 0.1% tazarotene gel daily for 6–8 months, achieving complete clinical and histological remission, with no recurrence observed during the follow-up period of 18 to 30 months. They concluded that tazarotene may be an alternative treatment option for certain patients with LM [80]. A randomized controlled trial conducted by Mark A. and colleagues assessed the efficacy of imiquimod 5% cream alone versus its use in combination with 0.1% tazarotene gel in treating LM. The results showed that the combination therapy increased the complete remission rate by 14% [81]. There are currently multiple ongoing clinical trials using a combination of imiquimod and tazarotene for treating melanoma in situ, and related results have been summarized in the existing literature [10]. These studies suggest that imiquimod alone or in combination with tazarotene is effective in treating LM and lentigo-malignant melanoma, with some patients achieving complete clinical and histological remission. However, the variability in efficacy may depend on the treatment protocol, patient differences, and lesion characteristics.

## 4. Tazarotene’s Mechanism of Action in Melanoma

The mechanism by which tazarotene inhibits melanoma growth may be related to the genes it regulates (Table 1).

*TIG1* is an RA-induced tumor suppressor gene [54,88,89], the expression of which is typically downregulated in melanoma [82,84]. *TIG1* inhibits melanoma growth through multiple mechanisms. First, it inhibits the mTOR signaling pathway via an associated regulator of PIKfyve (ArPikfyve, named VAC14). The mTOR signaling pathway plays a key role in cell growth, proliferation, and metabolism, and its abnormal activation is closely associated with the development of various cancers [90,91,92]. *TIG1* suppresses mTOR activity by interacting with VAC14, thereby inhibiting the growth and proliferation of melanoma cells [82]. Secondly, *TIG1* expression induces endoplasmic reticulum (ER) stress, leading to melanoma cell death. ER stress is triggered by the accumulation of misfolded proteins within cells, and prolonged ER stress can lead to apoptosis. *TIG1* promotes melanoma cell death by triggering ER stress and exerting antitumor effects [83]. Additionally, comprehensive bioinformatics analysis and experimental validation have suggested that *TIG1* could serve as a promising biomarker for cutaneous melanoma. Its expression level is closely related to the onset and progression of melanoma, indicating its potential value in diagnosis and prognostic assessment [84]. In summary, *TIG1* suppresses melanoma growth by, for example, inhibiting the mTOR signaling pathway and inducting ER stress.

*TIG2* (also known as chemerin, gene name *RARRES2*) plays an important immune regulatory role in melanoma suppression, primarily by recruiting and activating immune cells to exert antitumor effects [93,94]. Russell showed that *TIG2* effectively recruits and activates natural killer (NK) cells by binding to their receptor CMKLR1, enhancing their cytotoxicity against melanoma cells and inhibiting tumor growth [85]. Further research by Yan Song demonstrated that ATRA can partially achieve its antitumor effects by upregulating *TIG2* expression and relying on CMKLR1-mediated NK cell recruitment, revealing *TIG2*’s important role as an intermediary in the RA antitumor mechanism [86]. A study by Romain Ballet found that *TIG2* not only mobilizes NK cells but also promotes the migration of a CD8^+^ T cell subset with NK-like functionality; this plays a crucial role in immune surveillance in the tumor microenvironment, further enhancing *TIG2*’s antitumor effects [95]. Additionally, Jingjin Ma et al. showed that *TIG2* is involved in the regulation of tumor-associated macrophages (TAMs) within the tumor microenvironment. This “lock-and-key” interaction may help alter the tumor microenvironment and inhibit proliferation and metastasis [96]. In summary, *TIG2* primarily regulates the recruitment and activation of immune cells, including NK cells, CD8^+^ T cells, and TAMs, suppressing melanoma and having a synergistic effect with the RA pathway. These findings suggest that *TIG2* is not only a critical regulator of melanoma progression but also a potential therapeutic target, offering new avenues for immunotherapy and combination therapy with retinoid drugs for melanoma treatment.

*TIG3* is also a RA-induced tumor suppressor gene [97,98,99,100]. Its expression is downregulated in various cancers, including skin cancer and oral squamous cell carcinoma [50,101,102,103]. The long noncoding RNA HCP5, a tumor-suppressive molecule, regulates *TIG3* expression by binding to miR-12. High HCP5 expression upregulates *TIG3*, thus inhibiting the development of cutaneous melanoma [87]. In oral squamous cell carcinoma, *TIG3* expression correlates with tumor differentiation, and high expression induces cell differentiation and apoptosis, inhibiting invasive tumor growth [101]. *TIG3* expression is reduced in both skin cancer and psoriasis. Restoring its expression has been shown to inhibit abnormal keratinocyte proliferation, highlighting its essential role in maintaining normal skin cell proliferation [50]. In conclusion, *TIG3* inhibits melanoma growth through, for example, gene regulation, promoting cell differentiation and apoptosis, and inhibition of cell proliferation. Thus, restoring or enhancing *TIG3* expression may be a potential strategy for melanoma treatment.

As outlined by the functions of the above genes, tazarotene inhibits melanoma growth through the combined action of *TIG1*, *TIG2*, and *TIG3*. *TIG1* suppresses melanoma cell growth and proliferation by inhibiting the mTOR signaling pathway and inducing endoplasmic reticulum stress, which promotes cell death and exerts an anti-tumor effect. *TIG2* boosts immune surveillance by regulating the activation of immune cells, particularly the recruitment of NK cells and CD8^+^ T cells, thus inhibiting tumor growth. Additionally, TIG2 regulates TAMs, alters the tumor microenvironment, and further suppresses melanoma proliferation and metastasis. *TIG3* inhibits melanoma growth by regulating gene expression, promoting cell differentiation and apoptosis, and inhibiting cell proliferation. In conclusion, tazarotene exerts its anti-melanoma effects through multiple mechanisms, including inhibiting tumor cell growth, enhancing immune responses, and modulating the tumor microenvironment. *TIG1*, *TIG2*, and *TIG3* play key synergistic roles in this process. Therefore, restoring or enhancing the expression of these genes may represent a promising strategy for future melanoma treatments.

Tazarotene can regulate the growth of melanoma cancer cells. In addition to its direct effects on cancer cells, changes in the tumor microenvironment play an important role. IL-6 plays a complex, dual role in melanoma initiation, progression, and treatment response [104,105]. Armstrong et al. noted that, in some cases, the IL-6 secreted by melanoma cells could inhibit tumor growth in vivo, possibly because of its antitumor effects on immune regulation [106]. However, research by Lise Hoejberg indicated that IL-6 generally exhibits a protumor effect in patients with melanoma, particularly during advanced or metastatic stages [107]. Further investigation by Linnskog revealed that IL-6 enhances the migratory and invasive abilities of melanoma cells by upregulating WNT5A expression via the p38α-mitogen-activated protein kinase pathway, thereby promoting tumor metastasis and spread [108]. Additionally, Hoejberg et al. confirmed that elevated plasma IL-6 levels are associated with poor prognosis in patients with metastatic melanoma and can serve as an independent prognostic biomarker [109]. Meanwhile, a study by Wang and colleagues highlighted that IL-6 not only serves as a predictive marker for prognosis but also plays a significant predictive role in ICI therapy. High IL-6 levels are often associated with poor immune therapy responses, suggesting that it mediates immune suppression by promoting MDSC and TAM activity, leading to resistance to ICI treatment [110]. Therefore, IL-6 plays a dual role in melanoma; it can have antitumor effects in some cases but tends to promote tumor cell survival and invasion during disease progression or metastasis. Moreover, IL-6 serves as a serum biomarker of significant clinical value for melanoma prognosis and predicting immune therapy response. Targeting IL-6 or its signaling pathways, such as by using IL-6 receptor antagonists, may provide new therapeutic strategies for improving clinical outcomes for patients with melanoma [110,111,112]. According to recent studies, RA and its analogs inhibit IL-6 production in certain cell types. For example, RA suppresses IL-6 production in human lung fibroblasts induced by IL-1 [113]. The inhibitory effect on IL-6 production is mainly restricted to RAR receptor signaling and is related to the suppression of AP-1 and nuclear factor-IL-6 (NF-IL-6) activation [114]. Similarly, RAR receptor signaling can block the NF-IL-6 activation pathway in keratinocytes, affecting cell proliferation [115]. However, specific data on whether tazarotene directly inhibits IL-6 production in the melanoma tumor microenvironment are still lacking. Duvic et al. found that tazarotene inhibits IL-6 production in keratinocytes. Additionally, tazarotene may regulate immune responses by inhibiting AP1 transcription factor activity, suppressing T-cell activation, and reducing the production of IL-6 and other inflammatory molecules, such as ICAM-1 (Figure 2) [43,48].

## 5. Mechanisms of Drug Resistance and Changes in Retinoid Receptors in Melanoma: Implications for Development and Prognosis

The mechanisms of drug resistance in melanoma are complex and primarily involve genetic mutations in cancer cells, the tumor microenvironment, and cellular adaptability. Firstly, the common BRAF mutation in melanoma can lead to resistance to BRAF inhibitors. This occurs because tumor cells activate alternative signaling pathways, such as MAPK or PI3K/Akt, to bypass BRAF inhibition [116,117]. Additionally, cancer cells may undergo genetic mutations or epigenetic changes that alter drug entry or efflux mechanisms, reducing the effective drug concentration. Apart from genetic changes, the tumor microenvironment also plays a significant role in drug resistance. Immune evasion mechanisms, hypoxic conditions, and the presence of tumor-associated macrophages can all reduce treatment efficacy [118,119,120]. Finally, the high heterogeneity of tumor cells allows certain cells to survive during treatment and gradually proliferate, forming drug-resistant subpopulations. These mechanisms often result in melanoma exhibiting drug resistance during treatment, leading to failure.

**Figure 3:**
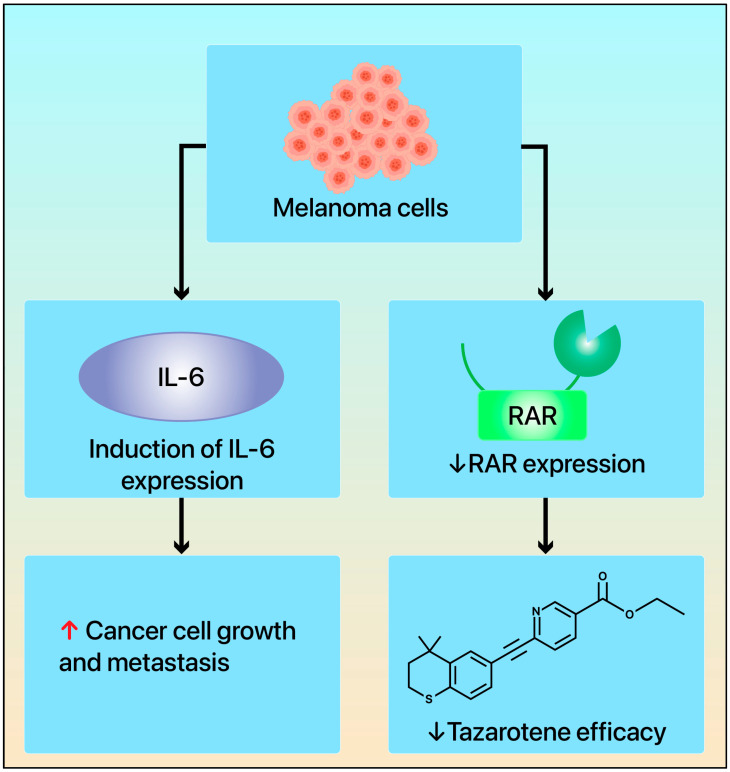
Changes in retinoid receptor signaling in melanoma development. When tazarotene binds to retinoid receptors, it induces the expression of *TIG1*, *TIG2*, and *TIG3* while inhibiting IL-6 expression to suppress MDSC activity. This process inhibits melanoma growth and promotes cancer cell death (Figure 2). However, when melanoma cells lose retinoid receptor expression, tazarotene loses its efficacy. Additionally, melanoma cells can induce IL-6 expression, which activates MDSC and promotes cancer cell growth and metastasis. TIG, tazarotene-induced gene; MDSC, myeloid-derived suppressor cell; IL-6, interleukin-6.

The mechanisms behind melanoma’s resistance to tazarotene or RA have not yet been fully elucidated. Nonetheless, genetic mutations in cancer cells leading to abnormal retinoid receptor expression may be one of the key factors. Chakravarti et al. explored changes in retinoid receptor (RARs and RXRs) expression in melanoma development and prognosis, along with potential mechanisms [121]. The study indicated that the expression of retinoid receptors, particularly RAR-β, RAR-γ, and RXR-α, is significantly reduced in some melanoma tissues. This reduction is closely associated with melanoma progression, invasiveness, and a poor clinical prognosis [79]. In normal skin cells, retinoid receptors maintain cellular homeostasis by regulating cell differentiation, proliferation, and apoptosis. However, in melanoma cells, the downregulation of these receptors leads to dysregulation in the RA pathway, causing abnormal cell proliferation and immune evasion. Additionally, the loss of RAR and RXR receptors may affect the expression of downstream genes, such as the tumor suppressor genes *TIG1* and *TIG3*, further promoting tumor cell growth and survival. Retinoid drugs (including tazarotene) can effectively inhibit tumor cell proliferation and promote cell differentiation and apoptosis by activating these receptors, thus demonstrating their potential therapeutic value. Therefore, the expression of retinoid receptors could serve as important biomarkers for melanoma prognosis and provide a theoretical foundation for the application of retinoid drugs in melanoma treatment (Figure 3). Future therapies aimed at restoring or activating retinoid receptors may become an important strategy to improve the prognosis of patients with melanoma.

Tazarotene has been approved by FDA for treating plaque psoriasis and acne vulgaris (Reference ID: 4249196). In clinical trials, it has been extensively studied for various dermatological conditions. For example, a Phase II clinical trial evaluated its efficacy in treating basal cell carcinoma and basal cell nevus syndrome (https://prevention.cancer.gov/clinical-trials/clinical-trials-search/nct00489086, accessed on 10 January 2025.). Additionally, research has explored the combination of tazarotene with microneedling for acne scar treatment, showing significant improvement [122,123,124]. Nonetheless, there are currently no clinical trials specifically investigating tazarotene as a standalone treatment for localized melanoma. Previous small-scale studies have examined its potential use alone or in combination with Imiquimod for localized melanoma treatment [10,80,81], but due to limited sample sizes and inconsistent efficacy, its clinical benefits remain unconfirmed. Given that melanoma progression involves various genetic alterations, further research into retinoid receptor expression and MAPK signaling pathway mutations in melanoma cells may help develop more precise treatment strategies.

## 6. Conclusions

Tazarotene is a third-generation retinoid derivative that has been widely used to treat skin diseases, such as psoriasis and acne. Recent studies have shown its potential inhibitory effects on melanoma cells. Its primary mechanism of action involves selectively activating RARs (RAR-β and RAR-γ), which further regulate the expression of downstream induced genes, such as *TIG1*, *TIG2*, and *TIG3*, thereby influencing the proliferation, differentiation, apoptosis, and immune modulation of melanoma cells in the tumor microenvironment. *TIG1* promotes tumor cell apoptosis and reduces abnormal cell proliferation by inhibiting the mTOR signaling pathway and inducing an ER stress response. *TIG2* plays an important role in the melanoma immune microenvironment by regulating the recruitment of NK and CD8^+^ T cells via the CMKLR1 receptor, thereby enhancing the ability of the immune system to eliminate tumor cells. Decreased *TIG3* expression is closely related to high invasiveness and poor prognosis in melanoma. Tazarotene restores *TIG3* expression, further regulating cell proliferation and differentiation to inhibit tumor cell growth and spread. In addition, tazarotene demonstrates synergistic effects in immune modulation within the tumor microenvironment, not only by directly inhibiting tumor cell proliferation but also by indirectly promoting the activation and antitumor functions of immune cells. These multilayered molecular mechanisms reveal the complex role of tazarotene in melanoma treatment and provide a scientific basis for its future use as an adjunct or local treatment strategy. However, most studies are still in the cellular and animal experimental stages, and more clinical trials are needed to verify its safety and efficacy for melanoma treatment. This review aimed to systematically explore the gene regulation mechanisms of tazarotene and its effects on melanoma cell growth, further uncovering its potential value in cancer therapy and providing theoretical support for future clinical applications.

## Figures and Tables

**Table 1:** Putative function of novel genes regulated by tazarotene in melanoma.

Gene	Function	References
*TIG1*	Regulation of mTOR signaling and ER stress response	[82,83,84]
*TIG2*	Recruitment of NK and CD8^+^ T cells	[85,86]
*TIG3*	Inducing of cell differentiation and death	[50,87]

*TIG*, tazarotene-induced gene; ER, endoplasmic reticulum; NK, natural killer.

## Abbreviations

The following abbreviations are used in this manuscript:

TIG1	tazarotene-induced gene-1
RA	retinoic acid
ATRA	all-trans RA
RARs	retinoic acid receptors
RXRs	retinoid X receptors
IL-6	interleukin-6
MDSCs	myeloid-derived suppressor cells
LM	lentigo maligna
ER	endoplasmic reticulum
NK	natural killer
TAMs	tumor-associated macrophages
ICI	immune checkpoint inhibitor
